# Extracellular perception of multiple novel core effectors from the broad host-range pear anthracnose pathogen *Colletotrichum fructicola* in the nonhost *Nicotiana benthamiana*

**DOI:** 10.1093/hr/uhae078

**Published:** 2024-03-14

**Authors:** Mengqing Han, Chunhao Wang, Wenhui Zhu, Yuemin Pan, Lili Huang, Jiajun Nie

**Affiliations:** Anhui Province Key Laboratory of Crop Integrated Pest Management, Anhui Agricultural University, Hefei 230036, China; Anhui Province Key Laboratory of Crop Integrated Pest Management, Anhui Agricultural University, Hefei 230036, China; Anhui Province Key Laboratory of Crop Integrated Pest Management, Anhui Agricultural University, Hefei 230036, China; Anhui Province Key Laboratory of Crop Integrated Pest Management, Anhui Agricultural University, Hefei 230036, China; State Key Laboratory for Crop Stress Resistance and High-Efficiency Production, College of Plant Protection, Northwest A&F University, Yangling, Shaanxi 712100, China; Anhui Province Key Laboratory of Crop Integrated Pest Management, Anhui Agricultural University, Hefei 230036, China

## Abstract

*Colletotrichum fructicola* is emerging as a devastating pathogenic fungus causing anthracnose in a wide range of horticultural crops, particularly fruits. Exploitation of nonhost resistance (NHR) represents a robust strategy for plant disease management. Perception of core effectors from phytopathogens frequently leads to hypersensitive cell death and resistance in nonhost plants; however, such core effectors in *C. fructicola* and their signaling components in non-hosts remain elusive. Here, we found a virulent *C. fructicola* strain isolated from pear exhibits non-adaptation in the model plant *Nicotiana benthamiana*. Perception of secreted molecules from *C. fructicola* appears to be a dominant factor in NHR, and four novel core effectors—CfCE4, CfCE25, CfCE61, and CfCE66—detected by *N. benthamiana* were, accordingly, identified. These core effectors exhibit cell death-inducing activity in *N. benthamiana* and accumulate in the apoplast. With a series of CRISPR/Cas9-edited mutants or gene-silenced plants, we found the coreceptor BAK1 and helper NLRs including ADR1, NRG1, and NRCs mediate perceptions of these core effectors in *N. benthamiana*. Concurrently, multiple *N. benthamiana* genes encoding cell surface immune receptors and intracellular immune receptors were greatly induced by *C. fructicola*. This work represents the first characterization of the repertoire of *C. fructicola* core effectors responsible for NHR. Significantly, the novel core effectors and their signaling components unveiled in this study offered insights into a continuum of layered immunity during NHR and will be helpful for anthracnose disease management in diverse horticultural crops.

## Introduction

Plants are threatened by numerous phytopathogens, causing significant damage to annual agricultural food production. Though plants can be infected by adapted pathogens, they have evolved a multilayered immune system to defend against them [1, 2]. Notably, this immune system also enables plants to be completely resistant to non-adapted microbes in the nature, the phenomenon of which is called nonhost resistance (NHR) [3, 4]. Given NHR is robust, broad spectrum and durable, exploiting NHR represents a promising way of disease management in agriculture.

To successfully infect a plant, adapted pathogens must first overcome physiochemical barriers and then subvert the plant immune system by producing various effectors [4–6]. In response, the plant activates immune responses by recognizing pathogen-associated molecular patterns (PAMPs) through cell surface-localized pattern recognition receptors (PRRs), which are either receptor-like kinases (RLKs) or receptor-like proteins (RLPs), or by detecting effectors through nucleotide-binding leucine-rich repeats (NLRs) in the cytoplasm [1, 7]. PAMP recognition amounts pattern-triggered immunity (PTI), while effector recognition leads to a more robust effector-triggered immunity (ETI), typically accompanied by localized plant cell death. For some non-adapted pathogens, their effector genes are not usually activated and ETI does not occur, which accounts for a determinant of NHR [4, 5]. Regardless of this, NHR is considered analogous to host plant resistance, encompassing PTI and ETI [4]. Although both components contribute to NHR, PTI is proposed to play a primary role in non-host plants that are distantly related to natural hosts, with ETI being more prevalent in closely related non-host plants [8]. However, there is limited evidence supporting this hypothesis to date.

A few components have been illustrated to be central regulators of plant immunity. The leucine-rich repeat (LRR) RLKs BRI1-Associated Kinase-1 (BAK1) and Suppressor of BIR1–1 (SOBIR1) act as two essential co-receptors in PTI [9, 10]. Both BAK1 and SOBIR1 associate with PRRs for immune signaling transduction following PAMP perception. Upon ligand binding, BAK1 could be recruited by RLKs to form a bipartite receptor complex, whereas SOBIR1 constitutively associates with RLPs and then associates with BAK1 to form a tripartite complex [11, 12]. With regard to ETI, ENHANCED DISEASE SUSCEPTIBILITY (EDS1) among nucleocytoplasmic lipase-like proteins stands out as a typical controller of NLR-mediated immunity [13–15]. Another well-known regulator of ETI falls into ‘helper’ NLRs (hNLRs). Regularly, NLRs can be classified into two major sub-groups based on their N-terminus: TNLs with a Toll-interleukin 1 receptor (TIR) domain and CNLs with a coiled coil (CC) domain [16, 17]. Some CNLs, featuring a conserved RESISTANCE TO POWDERY MILDEW 8 (RPW8) at the N-terminus, are referred to as RNLs [18]. While most TNLs and CNLs function as sensor NLRs (sNLRs) during effector recognition, RNLs, also termed hNLRs, do not detect pathogens but operate downstream of diverse sNLRs as signaling hubs [16, 19]. Three families of hNLRs have been identified thus far: ACTIVATED DISEASE RESISTANCE 1 (ADR1), N Requirement Gene 1 (NRG1), and the Solanaceae-specific NLR required for cell death (NRC) [19–21]. Though these components are well characterized in plant immunity research, evidence for their roles in NHR is limited.

A central notion in plant immune system against microorganisms is perception of nonself factors, typically the aforementioned PAMPs and effectors. PAMPs are generally conserved signatures among different microbes; by contrast, effectors are usually species-specific [22, 23]. It is worth mentioning that the dichotomy for PAMP and effector is much blurred [24]. An increasing number of secreted effectors, like XEG1 from *Phytophthora sojae*, VmE02 from *Valsa mali*, and VdEIX3 from *Verticillium dahliae*, which are all widely distributed in microorganisms, have been established as PAMPs [25–27]. Either labeled PAMPs or effectors, these molecules that are conserved among different isolates or species are deemed ‘core effectors’ [28, 29]. Recent years witnessed the dissection of core effectors concerning NHR from multiple phytopathogens. For example, 57 core effectors of *Phytophthora infestans* were used to screen NLRs in the nonhost *Capsicum annuum*, resulting in identification of multiple functional NLRs that are more tolerant to effector suppression [30]. Additionally, 30 core effectors were identified in *Colletotrichum orbiculare*, and five of them collectively contribute to pathogen virulence in a host-selective manner [31]. This indicates certain component(s) in specific hosts may evade attacks from virulence core effectors, and characterization of these components may facilitate NHR exploitation. Thus, elucidation of pathogen core effectors is of great significance for gaining insights into NHR.

The *Colletotrichum* genus comprises nearly 600 species that cause severe damage on most crops, and as such it ranges in the top 10 fungal pathogens in phytopathology [32, 33]. Some *Colletotrichum* species exhibit a broad host range and can establish compatible interactions with model plants. For example, *Colletotrichum higginsianum* can successfully infect *Arabidopsis thaliana* (Brassicaceae), while *C. orbiculare* is capable of infecting *Nicotiana benthamiana* (Solanaceae) [34, 35]. *Colletotrichum fructicola* is a notorious pathogenic fungus that brings about devastating anthracnose in multiple plants, particularly fruit crops like apple, pear, peach, mango, avocado, and strawberry [36, 37]. In the past two decades, genome sequencing has largely accelerated characterization of pathogen effectors. The first sequenced *C. fructicola* genome was reported in 2013, with over 700 effectors predicted [38]. However, until recently, only a few effectors in *C. fructicola* have been characterized [39, 40]. The effector repertory of this fungus, especially the core effectors, remains quite elusive, not to mention their signaling components in plants.

In this study, we investigated in depth the core effectors from a virulent *C. fructicola* strain DSCF-02 isolated from pear (*Pyrus bretschneideri* Rehd.), followed by plant signaling components analysis using a series of CRISPR/Cas9-edited mutants of *N. benthamiana*. We found that *N. benthamiana* serves as a nonhost of DSCF-02, with conidia of DSCF-02 triggering strong NHR responses in *N. benthamiana*. Bioinformatics combined with agroinfiltration-mediated high throughput screening identified four uncharacterized core effectors (CfCE4, CfCE25, CfCE61, and CfCE66) that can induce cell death in *N. benthamiana*. These core effectors all require the BAK1 coreceptor and hNLRs (ADR1, NRG1, and NRCs) for cell death induction. Further analysis revealed that a stack of genes encoding PRRs and NLRs are activated upon *C. fructicola* detection. This study comprehensively characterized the repertory of *C. fructicola* core effectors for the first time and provided new insights into a PTI-ETI continuum during NHR, which could aid *C. fructicola* management through NHR utilization in future.

## Results

### *C. fructicola* DSCF-02 is a non-adapted pathogen on *N. benthamiana*

**Figure 1:**
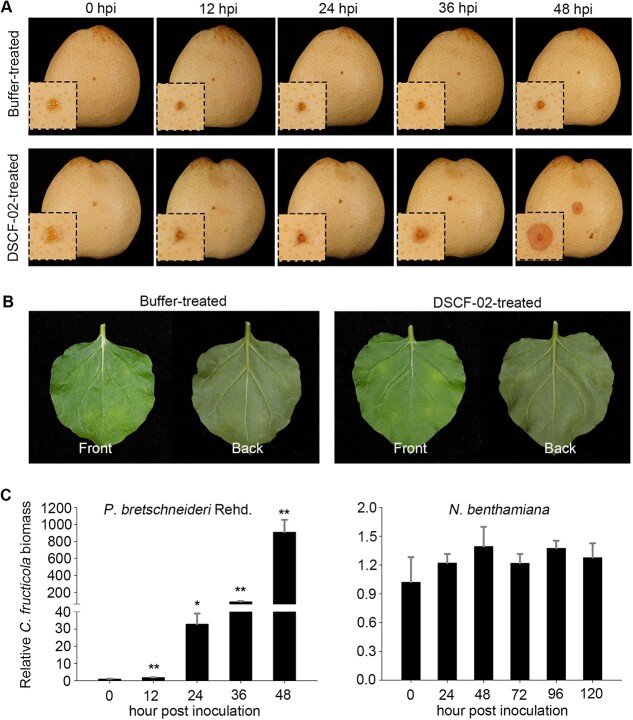
*Colletotrichum fructicola* is a non-adapted fungus in *Nicotiana benthamiana*. **A** Disease lesions of pear fruits after *C. fructicola* inoculations. Conidia of *C. fructicola* were inoculated on ‘Dangshansuli’ pear (*Pyrus bretschneideri* Rehd.). Conidia buffer was infiltrated as a negative control. Disease lesions were observed and photographed every 12 hours post inoculation (hpi) within 48 h. **B** Phenotype of *N. benthamiana* leaves after inoculation of *C. fructicola* conidia. The leaves were inoculated with *C. fructicola* conidia by infiltration, and photographs showing both the front and the back sides were taken at 120 hpi. **C** Relative fungal biomass after inoculation of *C. fructicola* on pear host and *N. benthamiana* nonhost. Relative biomass was quantified by transcription-quantitative polymerase chain reaction (RT-qPCR) to measure the ratios of *C. fructicola* to pear or *N. benthamiana* DNA. PbrTubulin and *NbActin* were used as the internal references in pear and *N. benthamiana*, respectively. Differences were determined with Student’s *t*-test. Values are shown as means ± SD. ^*^P < 0.05; ^**^P < 0.01.

With the aim to obtain the pathogen of pear anthracnose, we isolated a *Colletotrichum fructicola* strain DSCF-02 from diseased ‘Dangshansuli’ pear (*P. bretschneideri* Rehd.) (Fig. S1, see online supplementary material). Inoculation assays showed that DSCF-02 is a virulent strain that can cause anthracnose disease on multiple fruits including pear, apple, and peach (Fig. S1, see online supplementary material). For pear inoculation, apparent disease symptoms were detectable as early as 36 h post inoculation (hpi), and much larger lesions could be observed 48 hpi (Fig. 1A). To test whether DSCF-02 can infect the model plant *N. benthamiana*, fungal conidia were infiltrated into plant leaves. As was shown, compared to buffer-treated leaves, no disease symptom developed even till 120 hpi (Fig. 1B).

To further confirm the above results, we calculated the relative biomass of *C. fructicola* during the inoculation assays. It showed that *C. fructicola* biomass was dramatically elevated during pear infection, with a near 1000-fold increase at 48 hpi (Fig. 1C). For *N. benthamiana* inoculation, no obvious biomass changes were detected from 24 to 120 hpi (Fig. 1C), suggesting DSCF-02 fails to proliferate surrounding *N. benthamiana* cells. Collectively, these data revealed that *N. benthamiana* is a non-host for *C. fructicola* strain DSCF-02.

### DSCF-02 activates potent immune response in *N. benthamiana*

**Figure 2:**
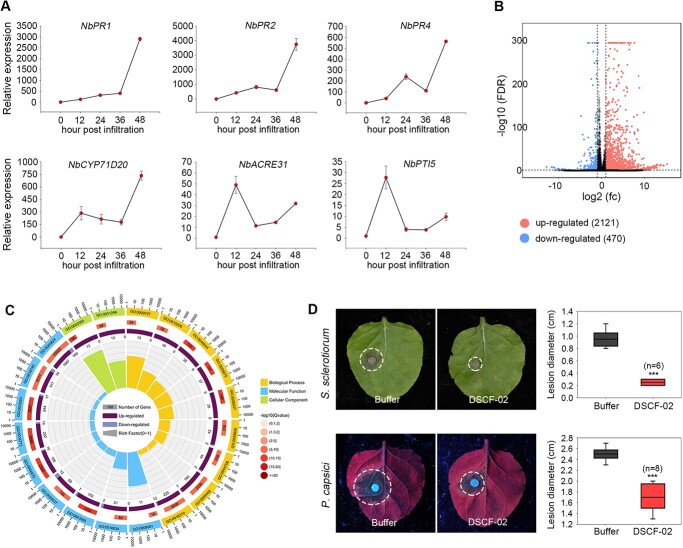
*Nicotiana benthamiana* recognizes *Colletotrichum fructicola* and induces strong immunity. **A** Transcript accumulation of *N. benthamiana* immune-related genes after inoculation with *C. fructicola* conidia determined by RT-qPCR. The conidia were infiltrated into plant leaves, and relative expression of immune-related genes including *NbPR1*, *NbPR2*, *NbPR4*, *NbCyp71D20*, *NbAcre31*, and *NbPTI5* were calculated from 12 to 48 hpi. *NbActin* was used as the internal reference. **B** Volcano plot of the differentially expressed genes (DEGs) between conidia*-* and buffer*-*infiltrated *N. benthamiana* samples. **C** Gene ontology (GO) classification of *N. benthamiana* transcriptome. Biological process, molecular function, together with cellular component were shown as three main GO categories. **D***Sclerotinia sclerotiorum*- and *Phytophthora capsici*-caused disease lesions in *N. benthamiana*. The leaves were pretreated with *C. fructicola* conidia by infiltration 12 h before pathogen inoculations. Conidia buffer was infiltrated as a negative control. Disease lesions of *S. sclerotiorum* were calculated at 24 hpi, and disease lesions of *P. capsici* were calculated at 36 hpi. Differences were determined with Student’s *t*-test. Values are shown as means ± SD. ^***^P < 0.001.

Because *N. benthamiana* inoculation was performed by infiltration, the physiochemical barriers like leaf cuticular layers were excluded*.* Hence, we speculated immune responses in *N. benthamiana* represent the major obstacle for this fungus. To test this hypothesis, a set of immune-related genes was transcriptionally analysed during *C. fructicola* inoculation of *N. benthamiana*, including genes encoding pathogenesis-related proteins (*NbPR1*, *NbPR2*, and *NbPR4*) and PTI marker genes (*NbPTI5*, *NbACRE31*, and *NbCYP71D20*). As a result, the transcripts of all tested genes were considerably induced from 12 to 48 hpi (Fig. 2A). Particularly, *NbPR1* and *NbPR2* were up-regulated by 3000- and 4000-fold 48 hpi, respectively.

**Figure 3:**
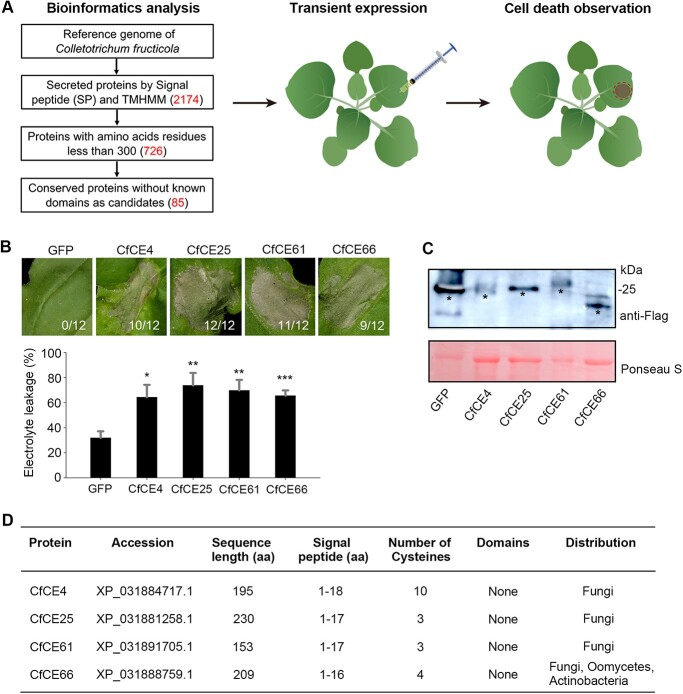
CfCE4, CfCE25, CfCE61, and CfCE66 were identified as novel core effectors of *Colletotrichum fructicola* that trigger *Nicotiana benthamiana* cell death. **A** Diagrammatic representation of pipeline and strategy for identification of core effectors of *C. fructicola*. **B** Phenotype of CfCE4-, CfCE25-, CfCE61-, and CfCE66-triggered cell death in *N. benthamiana*. Indicated genes were transiently expressed in *N. benthamiana* leaves via agroinfiltration. Representative leaves were photographed 4 d post agroinfiltration (dpa). The number of leaves showing the phenotypes (numerator) and that of total surveyed leaves (denominator) were indicated by numbers at the bottom. Cell death quantification was assessed by electrolyte leakage. Differences were determined with Student’s *t*-test. Values are shown as means ± SD. ^*^P < 0.05; ^**^P < 0.01; ^***^P < 0.001. **C** Western blotting detection of the transiently expressed proteins with anti-Flag antibody. Total proteins were stained with Ponceau S to show as the loading control. The bans with expected size are indicated by black asterisks. **D** Bioinformation of CfCE4, CfCE25, CfCE61, and CfCE66. The accession number, sequence length, predicted signal peptide (SP), number of cysteine residues, domains and distribution of homologues are shown.

For a molecular investigation of *N. benthamiana* NHR against *C. fructicola*, RNA-seq analysis was performed. It has been illustrated that *C. fructicola* establishes interactions with plants as early as 24 hours post inoculation [41, 42]; we therefore chose this time point for analysis. As was shown, 2591 differentially expressed genes (DEGs, 2-fold change) were identified in *N. benthamiana* (Fig. 2B; Table S1, see online supplementary material). Among these DEGs, 2121 genes were up-regulated and 470 genes were down-regulated. We further examined the functional roles of these DEGs by gene ontology (GO) enrichment analysis (Fig. 2C; Fig. S2, see online supplementary material). Some of the most enriched GO terms included ‘catalytic activity’ and ‘small molecular binding’ in the molecular functional group, ‘phosphorylation metabolic process’ and ‘protein phosphorylation’ in the biological process group, as well as ‘intrinsic component of membrane’ and ‘plasma membrane’ in the cellular component group. These data indicate that *N. benthamiana* NHR, to a great extent, attributes to molecular detections at plasma membrane.

To examine whether *C. fructicola*-triggered NHR could confer *N. benthamiana* resistance against phytopathogens, we inoculated the fungus *Sclerotinia sclerotiorum* and the oomycete *Phytophthora capsici* in *N. benthamiana* leaves pre-infiltrated with *C. fructicola* conidia. It showed that disease lesions caused by both pathogens were largely compromised in *C. fructicola* conidia-treated leaves, compared to those of buffer control-treated ones (Fig. 2D). The above results collectively demonstrated that *C. fructicola* DSCF-02 is detected by the *N. benthamiana* nonhost and induces strong plant immunity.

### Agroinfiltration-mediated screening identifies four novel core effectors that induce plant cell death

Plant immunity is often triggered via perception of pathogen-derived molecules. *C. fructicola-*triggered NHR on *N. benthamiana* may attribute to the fungus itself, given the existence of diverse elicitor components on fungal cell wall like chitin, glucans, and ergosterol [43–45]. Additionally, plant immunity activation can be ascribed to effector proteins secreted by phytopathogens. To determine which of them dominate NHR, boiled (killed) *C. fructicola* conidia (devoid of protein secretion ability) were infiltrated into *N. benthamiana* leaves for immunity induction tests, with normal conidia as the control. Intriguingly, the transcript levels of immune-related genes activated by boiled conidia were remarkably more attenuated than those of normal conidia (Fig. S3A, see online supplementary material). Similarly, pathogen inoculation assays found the boiled conidia-triggered resistance was greatly impaired (Fig. S3B, see online supplementary material). These data suggest secreted effectors of *C. fructicola* are major components that determine *N. benthamiana* NHR.

Because core effectors play crucial roles in NHR, we performed a *C. fructicola* genome research through bioinformatics analysis, and 85 conserved effector proteins without known domains were obtained as candidates (Fig. 3A; Table S2, see online supplementary material). These candidates were cloned and transiently expressed in *N. benthamiana*, with plant cell death as a hallmark for selection. We found four core effectors—CfCE4, CfCE25, CfCE61, and CfCE66—triggered cell death after transient expression in *N. benthamiana* (Fig. 3B). There was no cell death symptom observed in GFP-expressing leaves. Cell death activation was further confirmed by ion leakage quantification. Immunoblotting analysis revealed all these proteins were normally expressed (Fig. 3C).

**Figure 4:**
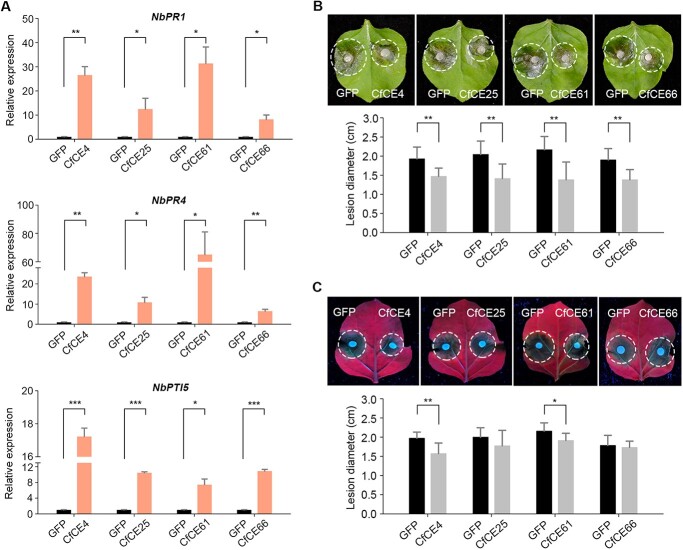
The four core effectors trigger *Nicotiana benthamiana* immunity. **A** Transcript accumulation of plant immune-related genes after transient expression the four core effectors. CfCE4, CfCE25, CfCE61, CfCE66, and GFP were transiently expressed in *N. benthamiana* leaves via agroinfiltration. Samples were taken 2 dpa, and relative expression of *NbPR1*, *NbPR4*, and *NbPTI5* were analysed by RT-qPCR. Differences were determined with Student’s *t*-test. Values are shown as means ± SD. ^*^P < 0.05; ^**^P < 0.01; ^***^P < 0.001. **B** Disease lesions of *N. benthamiana* caused by *Sclerotinia sclerotiorum*. The fungus was inoculated 24 h post agroinfiltration of CfCE4, CfCE25, CfCE61, CfCE66, and GFP in *N. benthamiana*. Disease lesions were calculated at 24 hpi. Differences were determined with Student’s *t*-test. Values are shown as means ± SD. ^**^P < 0.01. **C** Disease lesions of *N. benthamiana* caused by *Phytophthora capsici*. The pathogen was inoculated 24 h after transient expression of the core effectors. Disease lesions were calculated at 36 hpi. Differences were determined with Student’s *t*-test. Values are shown as means ± SD. ^*^P < 0.05; ^**^P < 0.01.

**Figure 5:**
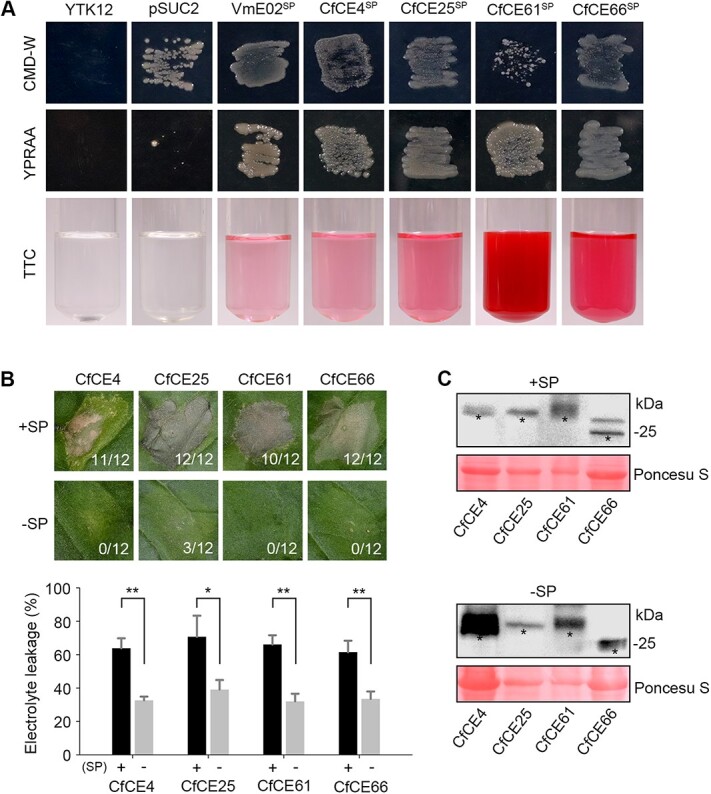
The core effectors are secretory and require signal peptides for cell death activation. **A** Validation of the signal peptides (SPs) of the core effectors with yeast secretion system. The YTK12 yeast strain, and the strain carrying an empty pSUC2 vector failed to grow on YPRAA media. In-frame fusion of the core effector SPs to the mature invertase allows for the invertase secretion, leading to successful growth on YPRAA media. The secreted invertase can also reduce 2,3,5-triphenyltetrazolium chloride (TTC) to red formazan. The SP of VmE02 was used as a positive control. **B** Phenotype of cell death triggered by the core effectors with or without corresponding SPs. Representative leaves were photographed at 4 dpa. The number of phenotype leaves (numerator) and that of total surveyed leaves (denominator) were indicated by numbers at the bottom of each leaf. Cell death quantification was assessed by electrolyte leakage. Differences were determined with Student’s *t*-test. Values are shown as means ± SD. ^*^P < 0.05; ^**^P < 0.01. **C** Western blotting detection of the transiently expressed proteins with anti-Flag antibody. Total proteins were stained with Ponceau S to show as the loading control. Black asterisks indicate the bans with expected size.

BLAST searches against the NCBI database illustrated that the four core effectors are widely spread in diverse microorganisms (Fig. 3D). Homologues CfCE4, CfCE25, and CfCE61 are distributed in multiple fungal species; nonetheless, homologues of CfCE66 can also be found in oomycetes and actinobacteria apart from fungi (Fig. 3D; Fig. S4, see online supplementary material). All core effectors contain a predicted signal peptide (SP) for protein secretion and certain numbers of cysteine residues that possibly form disulfide bridges for structural stabilization. Particularly, CfCE4 represents a small cysteine-rich protein with 10 cysteines in its mature sequence. Moreover, because no functional domains were identified in the four proteins (Fig. 3D), they represent novel core effectors that have not been characterized before.

### The four core effectors can activate plant immunity and promote resistance against filamentous phytopathogens

Given the capacity of *C. fructicola* to trigger NHR in *N. benthamiana*, we assayed whether CfCE4, CfCE25, CfCE61, and CfCE66 are able to activate *N. benthamiana* immunity. To this end, the four core effectors were separately agroinfiltrated into *N. benthamiana* leaves for transient expression, with GFP used as a negative control. It showed that the four core effectors transcriptionally activated three selected immune-related genes (*NbPR1*, *NbPR4*, and *NbPTI5*) without exception (Fig. 4A). In line with this, transient expression of the four core effectors obviously promoted *N. benthamiana* resistance against the fungus *S. sclerotiorum* (Fig. 4B). Additionally, CfCE4 and CfCE61 also significantly limited *P. capsici* infection of in *N. benthamiana* (Fig. 4C). Therefore, the four core effectors are most likely to be perceived by *N. benthamiana* cells, thereby triggering plant immunity.

### Signal peptide is indispensable for cell death-inducing activity of the four core effectors

To functionally validate the SPs of these core effectors, a yeast signal sequence trap system was used. It showed that the SPs of the four core effectors all enabled the secretion of invertase, leading to the normal growth of yeast on selective media (Fig. 5A). To confirm this result, we carried out a color reaction assay with 2,3,5-triphenyltetrazolium chloride (TTC), a chemical that can be reduced into an insoluble red-colored form in the presence of invertase. Consistently, we found the TTC solution unexceptionally turned red with the yeasts transformed with each SP (Fig. 5A). These findings demonstrate that the SPs of the core effectors can successfully guide protein secretion *in vivo*. Subsequently, we investigated whether the four core effectors require SPs to trigger plant cell death. For this, full-length proteins (+SP) and SP-deleted versions (-SP) were both transiently expressed in *N. benthamiana* leaves. Interestingly, while full-length proteins normally induced cell death, all SP-deleted versions lacked cell death-inducing activity (Fig. 5B). Western blotting detection showed the proteins were normally expressed (Fig. 5C). These results demonstrate SP is indispensable for cell death-inducing activity of the four effectors and suggest they probably function in the extracellular space of plant cells. We then isolated the apoplastic fluid from agroinfiltrated leaves and analysed protein accumulations via western blotting. As expected, full-length proteins but not those SP-deleted versions were detected in the apoplastic fluid (Fig. S5, see online supplementary material), confirming their extracellular localization.

**Figure 6:**
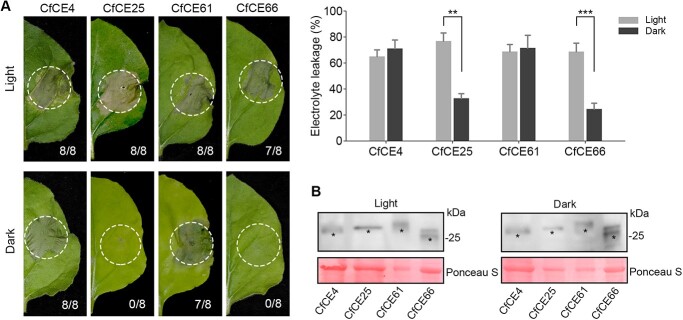
Light dependence of the core effectors. **A** Phenotype of the core effectors-triggered cell death under light and dark conditions. CfCE4, CfCE25, CfCE61, and CfCE66 were agroinfiltrated in *Nicotiana benthamiana* and the plants were kept in a greenhouse under light or dark conditions. Representative leaves were photographed at 5 dpa. The number of phenotype leaves (numerator) and that of total surveyed leaves (denominator) were indicated by numbers. Cell death quantification was assessed by electrolyte leakage. Differences were determined with Student’s *t*-test. Values are shown as means ± SD. ^**^P < 0.01; ^***^P < 0.001. **B** Western blotting detection of the transiently expressed proteins with anti-Flag antibody. Total proteins were stained with Ponceau S to show as the loading control. Black asterisks indicate the bans with expected size.

Previous studies have shown that light frequently contributes to plant immunity and programed cell death activation [46, 47]. To assess light-dependence of the core effectors-triggered cell death, *N. benthamiana* leaves transiently expressing CfCE4, CfCE25, CfCE61, and CfCE66 were kept in a greenhouse under light (day-night cycle) or dark conditions for five days. Consequently, CfCE4 and CfCE61 retained cell death-inducing in darkness, whereas CfCE25 and CfCE66-triggered cell death was almost abolished (Fig. 6A). Our ion leakage assays corroborated this result. Western blotting detection showed all agroinfiltrated proteins were normally expressed and there was no apparent difference in protein accumulations, indicating light shows no effects on protein expression (Fig. 6B). These results indicate *C. fructicola* effector perceptions are differentially mediated by light.

### The core effectors-triggered cell death requires BAK1 and helper NLRs

**Figure 7:**
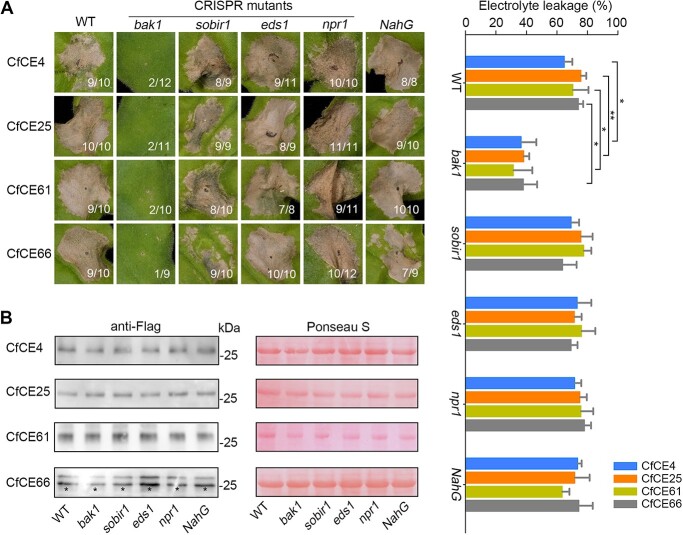
The core effectors depend on BAK1 for cell death activation in *Nicotiana benthamiana*. **A** Phenotype of cell death triggered by the core effectors in different mutants of *N. benthamiana*. CfCE4, CfCE25, CfCE61, and CfCE66 were transiently expressed in CRISPR/Cas9-edited *N. benthamiana* mutants including *bak1*, *sobir1*, *eds1*, and *npr1*, as well as a *NahG*-transgenic *N. benthamiana*. Cell death was observed and representative leaves were photographed at 4 dpa. The number of phenotype leaves (numerator) and that of total surveyed leaves (denominator) were indicated by numbers. Cell death quantification was assessed by electrolyte leakage. Differences were determined with Student’s *t*-test. Values are shown as means ± SD. ^*^P < 0.05; ^**^P < 0.01. **B** Western blotting detection of the transiently expressed proteins with anti-Flag antibody. Total proteins were stained with Ponceau S to show as the loading control. Black asterisks indicate the bans with expected size.

Given the central roles of BAK1, SOBIR1, and EDS1 in plant immunity regulation, we examined whether they are required for cell death-inducing activity of the core effectors. To this end, CfCE4, CfCE25, CfCE61, and CfCE66 were transiently expressed in *bak1* [48], *sobir1* [49], and *eds1* [50] mutants generated by CRISPR/Cas9 in *N. benthamiana*. Compared to the wild type (WT), the four core effectors retained the ability to trigger cell death in *sobir1* and *eds1*, but not *bak1* mutant (Fig. 7A). As salicylic acid (SA) serves as an essential hormone in plant immunity [51], we further test its involvement in cell death-inducing activity of the effectors. For this, two SA-impaired mutants, CRISPR/Cas9-edited *npr1* and *NahG*-transgenic *N. benthamiana* [52] were used for analysis. As indicated, obvious cell death can be observed in both of the two mutants (Fig. 7A). Western blotting analysis showed proteins were all normally expressed (Fig. 7B). These results suggest that SOBIR1, EDS1, and SA are dispensable for these core effectors-triggered cell death. However, the requirement of BAK1 indicates that unknown RLKs mediate their perceptions.

**Figure 8:**
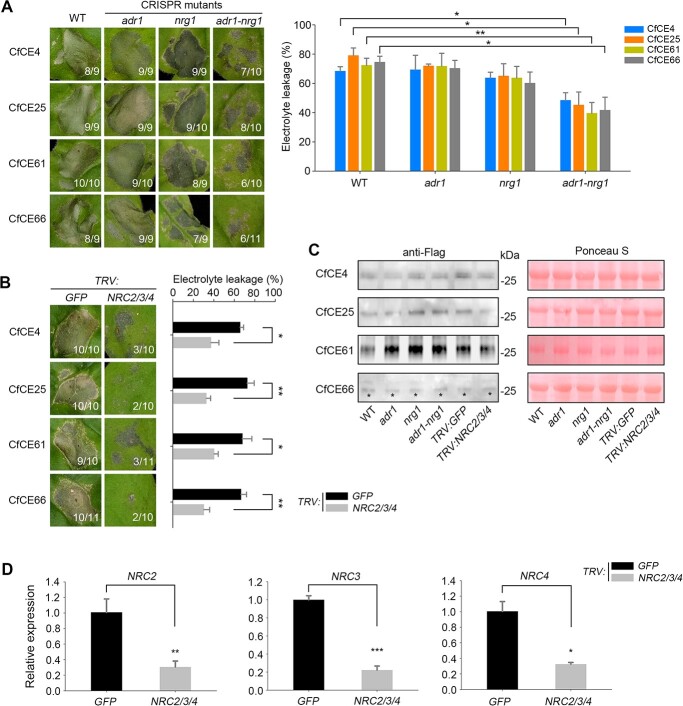
Helper NLRs are essential for the core effectors-triggered cell death in *Nicotiana benthamiana*. **A** Phenotype of the core effectors-triggered cell death in helper NLR mutants of *N. benthamiana*. CfCE4, CfCE25, CfCE61, and CfCE66 were transiently expressed in CRISPR/Cas9-edited *adr1*, *nrg1*, and *adr1*-*nrg1* mutants of *N. benthamiana*. Representative leaves were photographed 4 dpa. **B** Phenotype of cell death triggered by the core effectors in *NRCs*-silenced *N. benthamiana*. VIGS assays in *N. benthamiana* was conducted by infiltration of *Agrobacterium tumefaciens* carrying *TRV: NRC2/3/4* and *TRV: GFP* constructs. CfCE4, CfCE25, CfCE61, and CfCE66 were agroinfiltrated in gene-silenced *N. benthamiana* leaves. Representative leaves were photographed at 4 dpa. In **A** and **B**, the number of phenotype leaves (numerator) and that of total surveyed leaves (denominator) were indicated by numbers. Cell death was further quantified by electrolyte leakage. Differences were determined with Student’s *t*-test. Values are shown as means ± SD. ^*^P < 0.05; ^**^P < 0.01. **C** Western blotting detection of the transiently expressed proteins with anti-Flag antibody. Total proteins were stained with Ponceau S to show as the loading control. Black asterisks indicate the bans with expected size. **D** Silencing efficiency determination by RT-qPCR. Relative expression of *NRC2*, *NRC3*, and *NRC4* was normalized to *NbActin* and calibrated to the levels of *TRV: GFP-*infiltrated leaves. Differences were determined with Student’s *t*-test. Values are shown as means ± SD. ^*^P < 0.05; ^**^P < 0.01; ^***^P < 0.001.

In order to gain deeper insights into the signaling components participating in the perception of these core effectors, we subsequently focused on the helper NLRs (hNLRs), which have been demonstrated to be essential regulators of effector-triggered cell death [53, 54]. CRISPR/Cas9-edited *N. benthamiana* mutants includin*g adr1*, *nrg1*, as well as the double mutant *adr1-nrg1* were used for transient expression analysis. As indicated in Fig. 8A, the four core effectors exhibited significantly attenuated cell death-inducing activity in the *adr1-nrg1* double mutant but not *adr1* or *nrg1* mutants, in comparison with that of the WT (Fig. 8A). This suggests that ADR1 and NRG1 may redundantly mediate the core effectors’ perceptions. Furthermore, we employed virus-induced gene-silencing (VIGS) to silence the Solanaceae-specific NRCs (NRC2/3/4) for assays. Transient expression revealed that the core effectors all triggered a markedly compromised cell death on *NRCs*-silenced *N. benthamiana*, compared to the *GFP*-silenced plants (Fig. 8B). Western blotting analysis revealed that all proteins were normally expressed (Fig. 8C). Therefore, apart from ADR1 and NRG1, the NRC helpers also participate in perception of the four core effectors in *N. benthamiana*.

### Multiple PRR and NLR genes are activated by DSCF-02 in *N. benthamiana*

**Figure 9:**
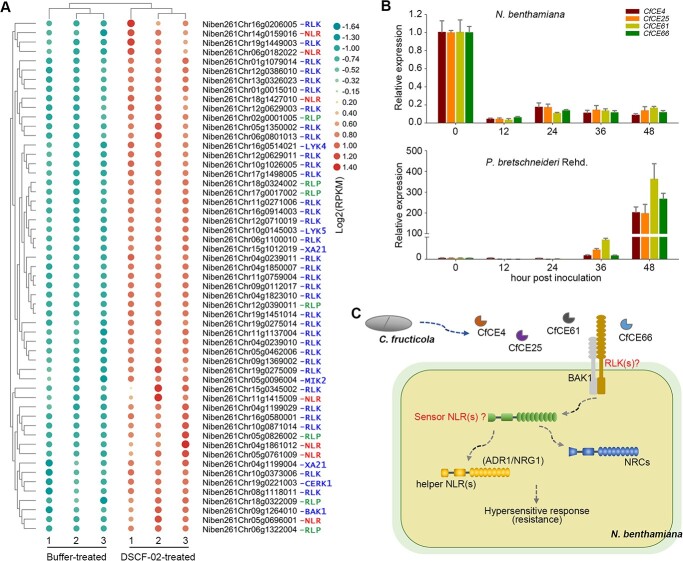
Multiple PRR and NLRs are involved in *Nicotiana benthamiana* perception of *Colletotrichum fructicola*. **A** Heatmap of 55 highly induced genes of *N. benthamiana* encoding PRRs and NLRs after *C. fructicola* inoculation. The heat map shows a double hierarchical cluster among DEGs (vertical) and samples (horizontal). **B** Expression profiles of *CfCE4*, *CfCE25*, *CfCE61*, and *CfCE66* after *C. fructicola* inoculation of *N. benthamiana* and pear. Transcript levels of the four genes at 0, 12, 24, 36, and 48 hpi of *C. fructicola* strain DSCF-02 were accessed by RT-qPCR, with *CfActin* used as the internal reference. **C** A proposed model for *N. benthamiana* detection of the non-adapted fungus *C. fructicola*. CfCE4, CfCE25, CfCE61, and CfCE66 represent four novel core effectors that can be recognized by the nonhost *N. benthamiana*. Unknown RLK(s) at plant cell surface may serve as PRR(s) that directly recognize these core effectors. Uncharacterized sensor NLRs (sNLRs), and the helper NLRs including ADR1, NRG1, and NRCs act as intracellular immune receptors that mediate perceptions of the core effectors. Such perceptions lead to hypersensitive response and disease resistance of the nonhost *N. benthamiana*.

Based on the indispensable roles of BAK1 and hNLRs in perception of the core effectors, we hypothesized that genes encoding pattern recognition receptors (PRRs) and NLRs may be differentially activated by *C. fructicola* during its interaction with the nonhost *N. benthamiana*. To test this hypothesis, RNA-seq data for DSCF-02-treated *N. benthamiana* were analysed. As a result, a list of 55 significantly induced genes encoding either PRRs or NLRs were obtained (Fig. 9A; Table S3, see online supplementary material). The majority of these genes (49 out of 55) encode PRRs. Among these PRRs, a large proportion (42 out of 49) are RLKs, and seven are RLPs. This observation aligns with our speculation that unidentified RLKs might serve as receptors for CfCE4, CfCE25, CfCE61, and CfCE66. Importantly, several well-characterized RLKs like BAK1, CERK1, LYK4, LYK5, and MIK2 were also up-regulated by DSCF-02 (Fig. 9A), suggesting recognition of diverse ligands like fungal chitin and phytocytokines [55–58] is accompanied during *N. benthamiana* perception of *C. fructicola*.

We next wondered whether the core effectors CfCE4, CfCE25, CfCE61, and CfCE66 are transcriptionally induced during *C. fructicola*–*N. benthamiana* interaction. Intriguingly, expression profile analysis illustrated that all of the four effectors were substantially down-regulated in all inoculated stages (Fig. 9B). We then assessed their expression patterns during the fungal infection of its pear host. The transcripts of these four genes remained unaltered among the first 24 hours of infection but were all enormously elevated in late infection stages (Fig. 9B). This result suggests that CfCE4, CfCE25, CfCE61, and CfCE66 may participate in pathogen virulence during host infections. However, they seem to be suppressed in the *N. benthamiana* nonhost. On the other hand, they can probably be perceived by undiscovered RLKs, with hNLRs serving as intracellular signaling partners, triggering hypersensitive cell death and *N. benthamiana* resistance against phytopathogens (Fig. 9C).

## Discussion

*C. fructicola* is a devastating fungus with broad host range, causing severe anthracnose of diverse plants, in particular the fruit crops [36, 37]. Exploiting NHR represents a promising strategy for efficient disease management due to its robustness, broad spectrum, and durability. This study delves into understanding the pathogen effectors and plant signaling components contributing to *N. benthamiana* NHR against *C. fructicola*. Notably, we identified four novel core effectors of *C. fructicola* and uncovered essential components for their recognition in the *N. benthamiana* nonhost.

There were observations over four decades ago that non-adapted pathogens trigger cell death at the infection site of nonhost plants and stop growing at a very early stage [59, 60]. With the research progress of plant immunity, it is known that pathogen-produced effectors perceived by plants serve as crucial elicitors of cell death. Particularly, an increasing number of core effectors (conserved effectors) have been shown to display cell death-inducing activity in plants [29, 61]. Our observation that *N. benthamiana* leaves infiltrated with *C. fructicola* conidia activated strong immune response suggests *N. benthamiana* can fully detect this fungus (Fig. 2). Because induced plant immunity was attenuated when the conidia were killed (Fig. S3A, see online supplementary material), we hypothesized that secreted effectors act as major determinants of *N. benthamiana* NHR. Taking all these into consideration, we analysed the genome of *C. fructicola* to obtain candidate core effectors, followed by agroinfiltrated-screening with cell death observation. Fortunately, four novel cell death-inducing core effectors—CfCE4, CfCE25, CfCE61, and CfCE66—were identified (Fig. 3B). The four core effectors were further shown to activate *N. benthamiana* immunity and disease resistance, which was consistent with the eliciting activity of *C. fructicola* conidia (Fig. 4). To be noted, CfCE25 and CfCE66 promoted *N. benthamiana* resistance against *S. sclerotiorum* but not *P. capsici*. It is possible that *P. capsici* produces oomycete-specific effectors, for example, RxLR effectors [62] that inhibit CfCE25- and CfCE66- but not the other two effectors-triggered immunity. *Zymoseptoria tritici* has been reported to produce a few cell death-inducing effectors recognized by the *N. benthamiana* nonhost [63], though it is undetermined whether these effectors are core effectors. It would be reasonable to speculate that cell death-inducing elicitors, either being core effectors or not, contribute largely to NHR against non-adapted pathogens.

PAMPs produced by microorganisms are conserved signatures sensed by plant cell-surfaced immune receptors in the extracellular space. Because the four identified core effectors all require SPs for cell death activation and accumulate in the apoplastic fluid (Fig. 5; Fig. S5, see online supplementary material), they are probably recognized as PAMPs by *N. benthamiana*. Consistently, we found their cell death-inducing activity depends on BAK1, a central immune coreceptor in plants (Fig. 7). SOBIR1 has been illustrated to be constitutively coupled with RLPs for immune signaling transduction upon PAMP perceptions [10, 11, 64]. The dispensable role of SOBIR1 in the four core effectors-triggered cell death (Fig. 7) implies RLPs are not PRRs of them. Therefore, CfCE4, CfCE25, CfCE61, and CfCE66 are most likely sensed by RLKs. Supporting this, 42 RLKs in *N. benthamiana* were significantly upregulated after treatment with *C. fructicola* conidia (Fig. 9A). As *N. benthamiana* is a solanaceous plant distantly related to the rosaceae *P. bretschneideri* Rehd, our findings support the hypothesis that PTI plays a major role in distantly related non-hosts. Identification of the RLKs responsible for their recognition will contribute to a molecular understanding of *N. benthamiana* NHR.

Apart from BAK1, we found that helper NLRs (hNLRs) including ADR1, NRG1, and the Solanaceae-specific NRCs are responsible for the core effectors-triggered cell death as well (Fig. 8), suggesting unidentified sensor NLRs (sNLRs) also mediate their recognitions. This is intriguing because it suggests PTI and ETI components are equally indispensable for recognition of these effectors by the nonhost *N. benthamiana*. From this perceptive, PTI and ETI seem to make undistinguishable contributions to NHR, regardless of the phylogenetic distance between host and nonhost plants. Actually, there is an intimate PTI-ETI continuum in the plant immune system, given their mutual potentiation of immune responses via cell surfaced and intracellular immune receptors [65–68]. Because NHR and host resistance are considered to be similar at the molecular level [4], it is not surprising that the PTI-ETI continuum occurs in NHR. EDS1, a crucial regulator of NLR-mediated resistance was shown to be dispensable for plant cell death triggered by the core effectors (Fig. 7). In *A. thaliana*, EDS1 exclusively dimers with PAD4 or SAG101 [15]. The EDS1-PAD4 and EDS1-SAG101 complexes further form heterodimers with ADR1 and NRG1, respectively [13, 14]. It is quite puzzling that the core effectors activated impaired cell death in *hNLRs-*mutants but not the *eds1* mutant (Figs 7 and 8). A possible explanation is that the EDS1-mediated immunity in *N. benthamiana* and *A. thaliana* may be diverged. The EDS1-PAD4-ADR1 module has been demonstrated to regulate PTI in *Arabidopsis* but seems to be dispensable for PTI in *N. benthamiana*, which can support this hypothesis to some degree [66, 69]. It is also intriguing that NRCs and the redundant ADR1/NRG1 helpers simultaneously participate in recognition of these core effectors, the result of which suggests there exists an intricate crosstalk between NRCs- and ADR1/NRG1-mediated signaling, though little evidence has been found thus far.

During expression profile analysis for the four core effectors, a distinct pattern emerged. The four effector genes remained suppressed during *C. fructicola*-*N. benthamiana* interaction but were considerably induced during fungal infection of the pear host (Fig. 9B). Possibly, because *C. fructicola* does not perceive *N. benthamiana* as a host, the effectors are therefore not necessarily expressed. By contrast, during *C. fructicola*-pear infection, these effectors might function as virulence factors and were significantly activated. Another possibility is that phytopathogens could selectively deploy effectors to infect hostplants, but during their attempted infection of nonhost plants, effector genes expression should be kept low to avoid over-activation of hypersensitive responses, thereby ensuring pathogen survival (Fig. 1c). Also, even though secreted effectors are probably the major determinant of *N. benthamiana* NHR against *C. fructicola*, CfCE4, CfCE25, CfCE61, and CfCE66 revealed in this study may play limited roles, considering their low expression during *C. fructicola*–*N. benthamiana* interaction. Because NHR is generally quantitatively inherited [4, 5], recognition of only a couple of effectors is not sufficient enough. In line with this, we found multiple PRRs and NLRs in *N. benthamiana* were induced by *C. fructicola* (Fig. 9A), implying perceptions of diverse ligands are involved in NHR. As we focused on novel effectors (without known domains) in this study, a large portion of effectors detected by *N. benthamiana* were inevitably excluded. Therefore, continuous efforts shall be made to illustrate *C. fructicola* PAMPs and effectors perceived by nonhost plants in future work.

In summary, this study has identified multiple novel core effectors from the non-adapted fungus *C. fructicola* recognized by *N. benthamiana* for the first time. The findings from this study advance our understanding of NHR and provide instructive insights for future disease management.

## Materials and methods

### Plants, strains, and their cultivation

*N. benthamiana* were grown in a greenhouse (16 h photoperiod, 22°C, 65% humidity). *C. fructicola* stain DSCF-02 was maintained on potato dextrose agar (PDA) (28°C). The bacterial strains *Escherichia coli* (Top10) and *Agrobacterium tumefaciens* (GV3101) were grown on lysogeny broth (LB) medium, at 37°C and 28°C, respectively.

### Plasmid construction

For transient expression, *C. fructicola* effector candidates were cloned from *C. fructicola* cDNA library with gene-specific primers (Table S4, see online supplementary material) using Hieff Canace® Plus High-Fidelity DNA Polymerase (Yeasen, Shanghai, China). These amplicons were ligated into the binary vector pCAMBIA1300 using Hieff Clone® Plus One Step Cloning Kit (Yeasen, Shanghai, China). To validate the secretory function of signal peptides (SP), corresponding sequences were amplified from the pCAMBIA1300 vectors, and were then introduced into pSUC2 vector. To perform VIGS, gene fragment amplifications were introduced into TRV2 vector using *N. benthamiana* cDNA library. All constructs were validated by sequencing in Generalbiol (Chuzhou, China).

### Agroinfiltration-mediated transient expression

*A. tumefaciens* strain GV3101 was transformed with the binary vectors by electroporation. Individual colonies were selected with antibiotics and further validated by PCR. A positive colony was picked for culturing in LB medium with a shaking incubator (220 rpm, 28°C) for 48 h. After centrifugation, the bacteria were resuspended in MES buffer (10 mM MgCl_2_, 200 μM acetosyringone, 10 mM 2-(N-morpholino) ethanesulfonic acid (MES), pH 5.7) for no less than 2 h in the dark. For transient expression, the suspended cells were diluted to a final OD_600_ of 0.8 in MES buffer, and were subsequently infiltrated into the back of *N. benthamiana* leaves using a needleless syringe. Cell death-inducing activities of transiently expressed proteins were monitored 3–6 d post agroinfiltration (dpa). For immunoblotting analysis, agroinfiltrated leaves were harvested into liquid nitrogen 2 dpa.

### Measurement of electrolyte leakage

To quantify effector-triggered cell death, ion leakage was measured as described [26]. In brief, six agroinfiltrated leaf disks were harvested with a cork-borer set (diameter 1 cm). The leaf disks were placed into a tube containing 4 mL distilled water and kept at room temperature (RT) for 5 h. The conductivity of this bathing solution was measured to produce ‘value A’ with an electroconductivity meter (Orion Lab Star EC112, Thermo Scientific, CA, USA). Subsequently, the tube with leaf disks was boiled for 15 min. After cooling to RT, the conductivity was again measured to generate ‘value B’. Ion leakage was shown as: (value A/value B) × 100%.

### Protein extraction and western blotting analysis

Lysis buffer (50 mM Tris, 150 mM NaCl, 0.5% TritonX-100, 1% proteinase inhibitor cocktail, 1 mM phenylmethanesulfonyl fluoride (PMSF), pH 7.5) was used to extract total proteins. Apoplastic fluid was extracted as described [70]. Protein samples were boiled in 5 × sodium dodecyl sulfate (SDS) loading buffer for 10 min before loading on a gel for electrophoresis. The proteins were subsequently transferred to a polyvinylidene difluoride (PVDF) membrane with transfer buffer. The membrane was then incubated with HRP-conjugated anti-Flag antibody (ABclonal, Wuhan, China) at RT for 3 h, and the blots were detected with Ultra High Sensitivity ECL Substrate Kit (Abcam, Cambridge, UK).

### Yeast signal sequence trap

A yeast secretion system was used to functionally validate the signal peptides (SPs) of effectors. Briefly, sequences encoding the SP of effectors were individually cloned into pSUC2 vectors that contain a truncated invertase lacking SP. Next, these vectors were transformed into the YTK12 yeast stain before screening on CMD-W medium plates (Coolaber, Beijing, China). Positive yeast colonies were chosen for invertase secretion analysis by cultivation on YPRAA medium plates (Coolaber, Beijing, China). To determine the enzymatic activity of invertase, an appropriate volume of yeast culture was pipetted into a glass tube containing 0.1% TTC solution (Coolaber, Beijing, China), and a colorimetric change was observed after incubation at 37°C for 10 min.

### RNA sequencing and RT-qPCR analysis

Conidia suspension of DSCF-02 and buffer (negative control) were infiltrated into healthy *N. benthamiana* leaves using a syringe without a needle. 24 h later, these leaves were harvested into liquid nitrogen. RNA sequencing of harvested samples was performed by Gene Denovo Biotechnology Co (Guangzhou, China). To perform RT-qPCR analysis, total RNA was extracted using MolPure® TRIeasy™ Plus Total RNA Kit (Yeasen, Shanghai, China), and cDNA synthesis was performed with Hifair® AdvanceFast 1st Strand cDNA Synthesis Kit (Yeasen, Shanghai, China). Assays were performed with SYBR Green Master Mix in a CFX96 Real-Time system. Relative expressions were calculated through the 2^-ΔΔCT^ method [71]. All primers used are listed in Table S4 (see online supplementary material).

### Virus-induced gene silencing

To perform TRV (tobacco rattle virus)-mediated gene silencing in *N. benthamiana*, TRV2 vectors were transformed into *A. tumefaciens* strain GV3101 via electroporation. Prior to injection, *A. tumefaciens* cultures carrying TRV2 vectors were mixed with those carrying TRV1 vector at 1:1 ratio in MES buffer. The mixed culture was adjusted to a final OD_600_ of 0.8 before agroinfiltration. Three weeks later, gene silencing efficiency was validated by RT-qPCR analysis, and successfully silenced plants were used for transient expression assays. The experiments were repeated no less than three times.

### Pathogen inoculation assays

*C. fructicola* strain DSCF-02 was inoculated on either ‘Dangshansuli’ pear or *N. benthamiana*. Conidia suspension of DSCF-02 was prepared by culturing fresh mycelial plugs (diameter 0.5 cm) in potato dextrose broth (PDB) medium on a shaking incubator (28°C, 220 rpm) for 4 d. Conidia were collected by filtering through a layer of Miracloth (Millipore). Then, collected conidia were washed three times in sterile distilled water and resuspended to a concentration of 1 × 10^7^ conidia/ml. For pear inoculation, 20 μL prepared conidial suspension was pipetted on wound-treated pear fruit, and the inoculated fruits were placed in a growth chamber at 25°C. For *N. benthamiana* inoculation, conidial suspension was infiltrated into healthy leaves using a needleless syringe. To determine disease progression, relative fungal biomass was calculated by RT-qPCR using reference genes *CfActin*, *PbrTubulin*, and *NbActin*.

To evaluate *N. benthamiana* resistance, the pathogenic fungus *S. sclerotiorum* strain 1980 and the oomycete *P. capsici* strain LT263 were used for inoculations. *S. sclerotiorum* and *P. capsici* were maintained on PDA plates and 20% (v/v) V8 juice agar plates, respectively, both at 25°C in the dark. Fresh mycelial plugs were collected from the plates and were inoculated on healthy *N. benthamiana* leaves. Inoculated leaves were lined in plastic trays in a growth chamber (25°C). Disease lesions were calculated 24 h post inoculation (hpi) for *S. sclerotiorum* and 36 hpi for *P. capsici.*

### Bioinformatics analysis

The reference genome of *C. fructicola* was obtained from the NCBI database. Secreted effector proteins were predicted online through the SignalP 6.0 server (https://services.healthtech.dtu.dk/services/SignalP-6.0/) and TMHMM 2.0 server (https://services.healthtech.dtu.dk/services/TMHMM-2.0/). Homologues of core effectors were obtained by querying their protein sequences against the NCBI database using BLAST searching programs, with a cut-off E-value of 1e-10. Phylogenetic dendrograms were constructed using MEGA X with maximum likelihood.

## Acknowledgements

We thank Prof. Yuanchao Wang from Nanjing Agricultural University for sharing the *bak1* mutant. We thank Prof. Hailong Guo from China Agricultural University for sharing the *eds1* mutant and *NahG*-transgenic plant that were originally from Prof. Jonathan D.G. Jones’s lab at the Sainsbury Laboratory. We also thank Prof. Xiangxiu Liang from South China Agricultural University for sharing the *npr1*, *adr1*, *nrg1*, and *adr1-nrg1* mutants. This work was financially supported by National Natural Science Foundation of China (32302301, U1903206) and the Talent Program of Anhui Agricultural University (rc342213).

## Author contributions

J.N. designed and conceived the research. J.N., M.H., C.W., and W.Z. performed the experiments. J.N., M.H., C.W., and W.Z. analysed the data. J.N., M.H., C.W., and W.Z. wrote the manuscript. Y.P. and L.H. critically reviewed this manuscript.

## Data availability

All relevant data can be found within the manuscript and its supporting materials.

## Conflict of interest statement

The authors declare no conflicts of interest.

## Supplementary data

Supplementary data is available at *Horticulture Research* online.

## Supplementary Material

Web_Material_uhae078
