# Supplementary material for: Extracellular perception of multiple novel core effectors from the broad host-range pear anthracnose pathogen Colletotrichum fructicola in the nonhost Nicotiana benthamiana
Source: Hortic Res. 2024 Mar 14;11(5):uhae078. doi: 10.1093/hr/uhae078 (PMC11101317; doi:10.1093/hr/uhae078)
Supplement: Web_Material_uhae078 [file web_material_uhae078.zip › Supplemental data.pdf]

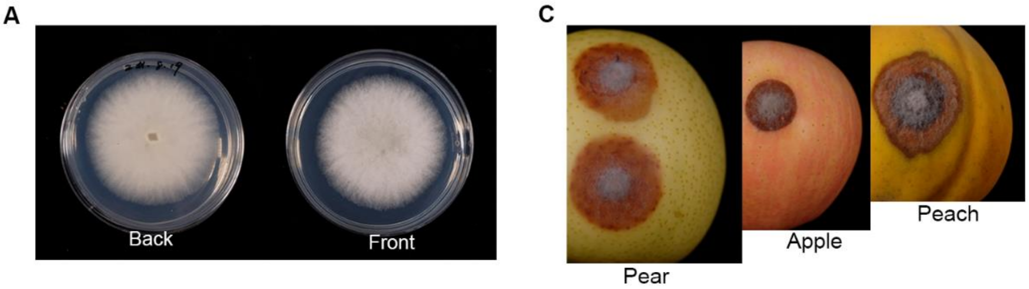


**B** Top 5 hits for ITS sequence of strain DSCF-02

| Strain/Isolate | Accession number | Sequence similarity |
|---|---|---|
| *Colletotrichum fructicola* PinkL3 | MT424894.1 | 100% |
| *Colletotrichum fructicola* PinkL2 | MT421924.1 | 100% |
| *Colletotrichum fructicola* CA-1 | MT393756.1 | 100% |
| *Colletotrichum fructicola* BL30 | MN273088.1 | 100% |
| *Colletotrichum fructicola* BL20 | MN273078.1 | 100% |

**Figure S1.** The *Colletotrichum fructicola* strain DSCF-02 is pathogenic on multiple fruits. **A** Filamentous growth of a fungus strain DSCF-02 isolated from diseased 'Dangshansuli' pear (*Pyrus bretschneideri* Rehd.) in Dangshan county, Anhui Province. Photographs were taken after cultivation on PDA plates for 4 d. Both the back and the front sides were shown. **B** DSCF-02 is a *C. fructicola* strain. Genomic DNA of DSCF-02 was extracted, and its internally transcribed space (ITS) region was sequenced and queried against the NCBI database. Best 5 hits of the resulting ITS sequences were listed. **C** Disease symptoms of pear, apple and peach inoculated with DSCF-02. Photographs were taken 3 d post inoculation.

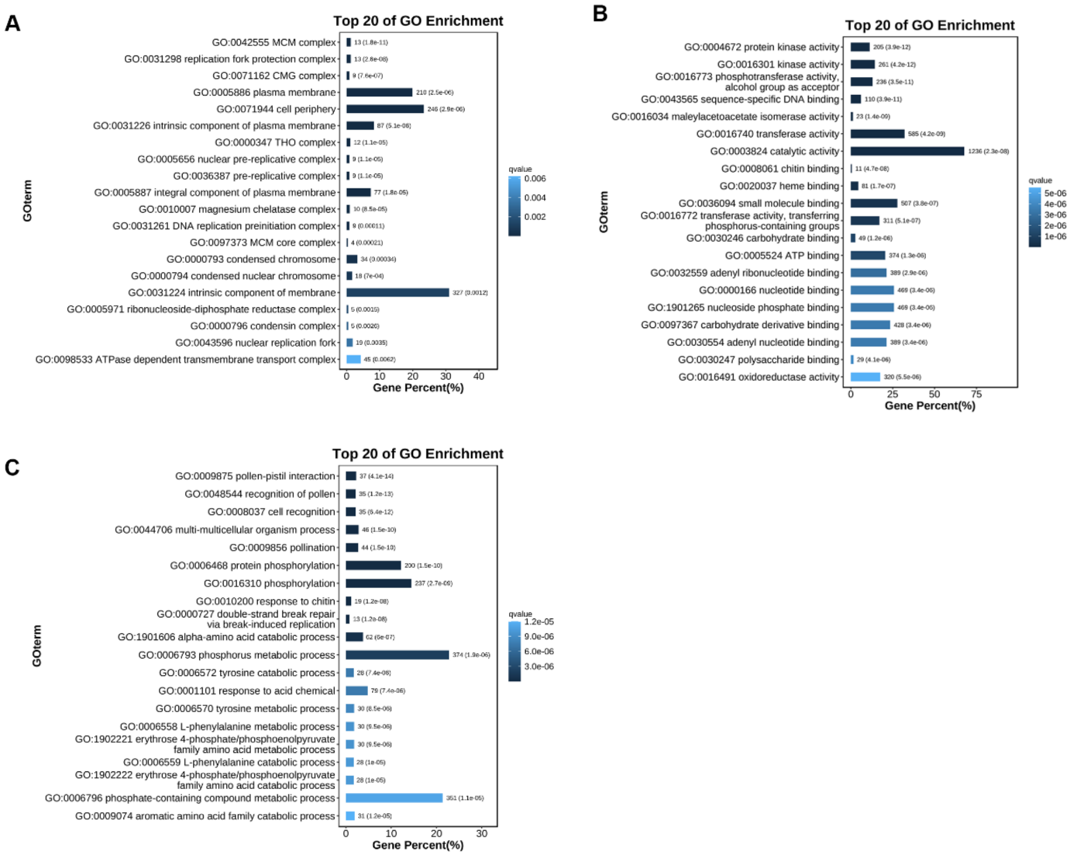


**Figure S2.** Gene ontology (GO) classification of *N. benthamiana* transcriptome treated with *C. fructicola* conidia. **A-C** The GO categories of cellular component (**A**), molecular function (**B**) and biological process (**C**). Top 20 of each GO enrichment were shown.

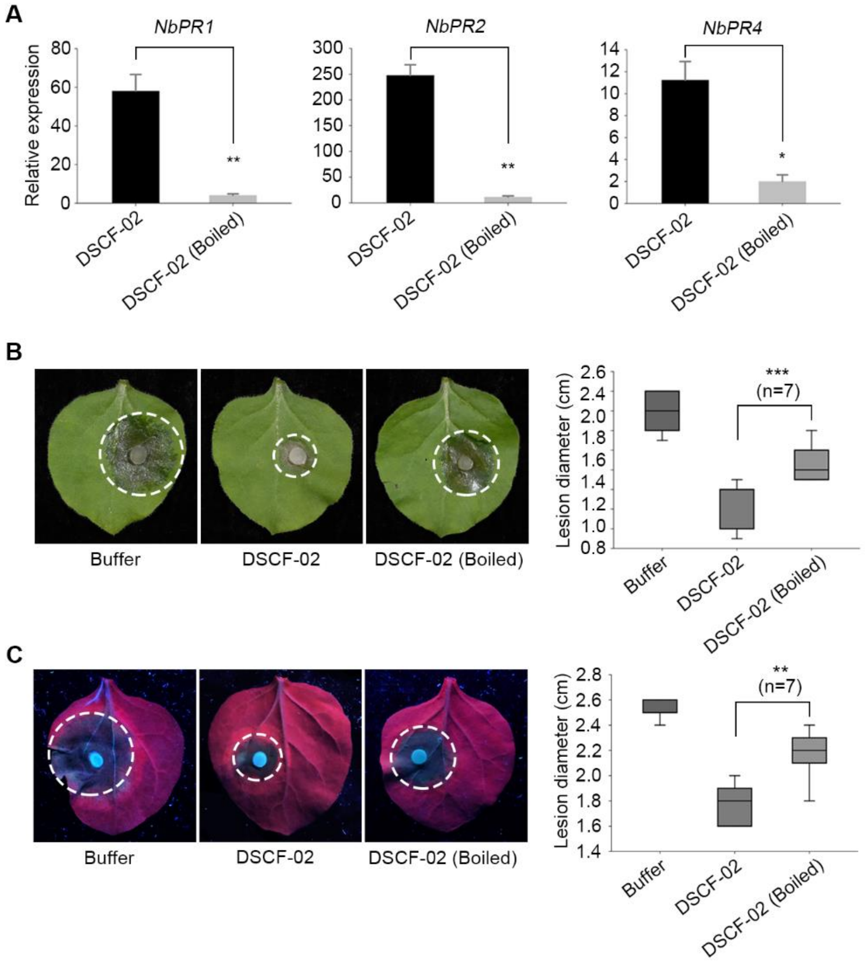


**Figure S3.** Boil-killed conidia of DSCF-02 show largely compromised immunity-eliciting activity in *N. benthamiana*. **A** Transcript accumulation of *NbPR1*, *NbPR2* and *NbPR4* in *N. benthamiana* after treatment with normal or boiled conidia of DSCF-02. The conidia were infiltrated into *N. benthamiana* leaves, and relative expression of tested genes was analyzed 6 h later by RT-qPCR. *NbActin* was used as the internal reference. Means and standard errors were calculated from three biological replicates. The statistical analyses were performed with Student's *t*-test. Bars indicate ± SD. *, $P < 0.05$; **, $P < 0.01$; ***, $P < 0.001$. **B-C.** Disease lesions of *N. benthamiana* caused by *Sclerotinia sclerotiorum* (**B**) and *Phytophthora capsica* (**C**). *N. benthamiana* leaves were infiltrated with normal or boiled *C. fructicola* conidia 12 h before pathogen inoculations. Conidia buffer was infiltrated as a negative control. Lesion diameters were calculated 24 hpi for *S. sclerotiorum* and 36 hpi for *P. capsica*. The statistical analyses were performed with Student's *t*-test. Bars indicate ± SD. **, $P < 0.01$; ***, $P < 0.001$.

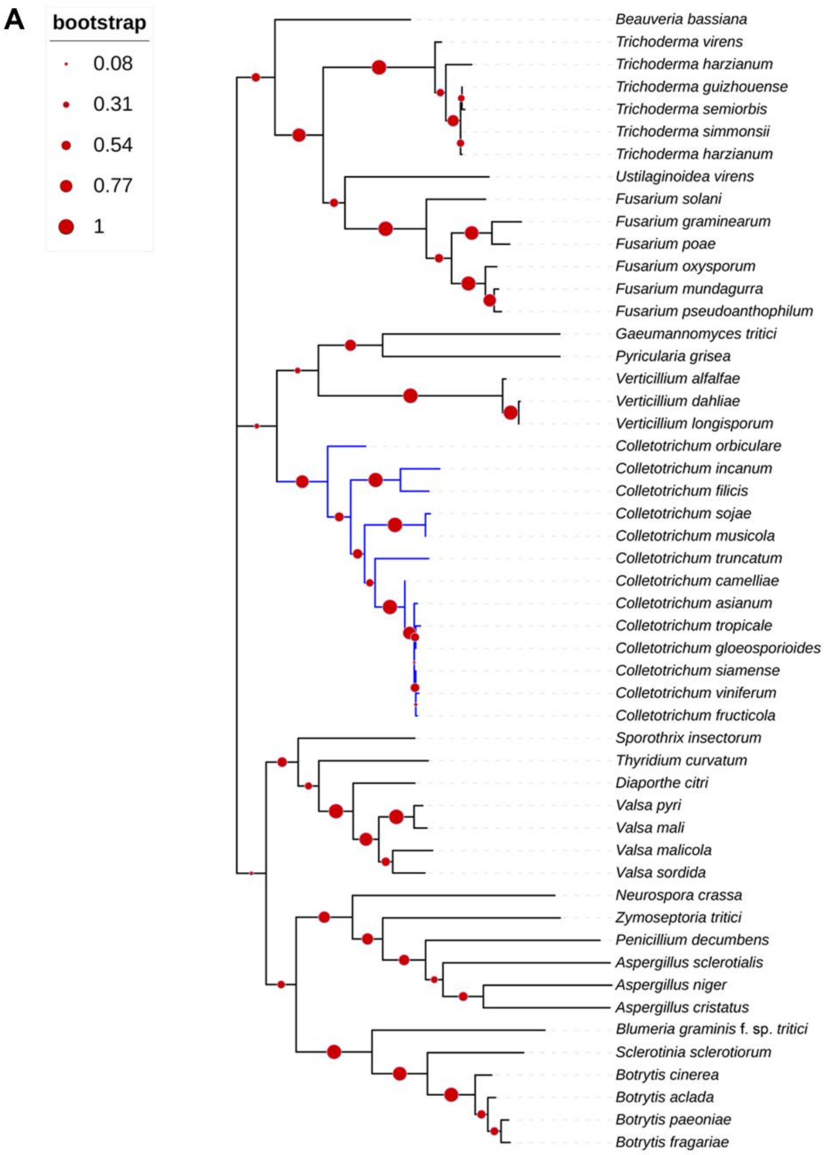
A
bootstrap
0.08
0.31
0.54
0.77
1
Beauveria bassiana
Trichoderma virens
Trichoderma harzianum
Trichoderma guizhouense
Trichoderma semiorbis
Trichoderma simmonsii
Trichoderma harzianum
Ustilaginoidea virens
Fusarium solani
Fusarium graminearum
Fusarium poae
Fusarium oxysporum
Fusarium mundagurra
Fusarium pseudoanthophilum
Gaeumannomyces tritici
Pyricularia grisea
Verticillium alfalfae
Verticillium dahliae
Verticillium longisporum
Colletotrichum orbiculare
Colletotrichum incanum
Colletotrichum filicis
Colletotrichum sojae
Colletotrichum musicola
Colletotrichum truncatum
Colletotrichum camelliae
Colletotrichum asianum
Colletotrichum tropicale
Colletotrichum gloeosporioides
Colletotrichum siamense
Colletotrichum viniferum
Colletotrichum fructicola
Sporothrix insectorum
Thyridium curvatum
Diaporthe citri
Valsa pyri
Valsa mali
Valsa malicola
Valsa sordida
Neurospora crassa
Zymoseptoria tritici
Penicillium decumbens
Aspergillus sclerotialis
Aspergillus niger
Aspergillus cristatus
Blumeria graminis f. sp. tritici
Sclerotinia sclerotiorum
Botrytis cinerea
Botrytis aclada
Botrytis paeoniae
Botrytis fragariae

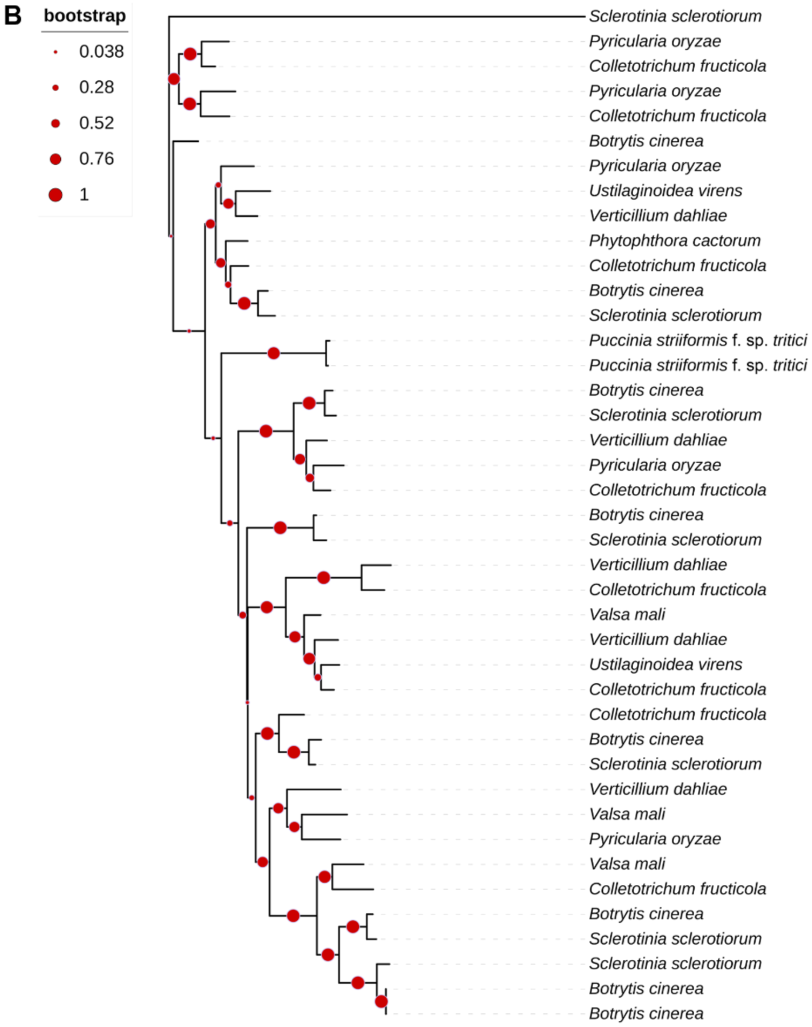
B
bootstrap
0.038
0.28
0.52
0.76
1
Sclerotinia sclerotiorum
Pyricularia oryzae
Colletotrichum fructicola
Pyricularia oryzae
Colletotrichum fructicola
Botrytis cinerea
Pyricularia oryzae
Ustilaginoidea virens
Verticillium dahliae
Phytophthora cactorum
Colletotrichum fructicola
Botrytis cinerea
Sclerotinia sclerotiorum
Puccinia striiformis f. sp. tritici
Puccinia striiformis f. sp. tritici
Botrytis cinerea
Sclerotinia sclerotiorum
Verticillium dahliae
Pyricularia oryzae
Colletotrichum fructicola
Botrytis cinerea
Sclerotinia sclerotiorum
Verticillium dahliae
Colletotrichum fructicola
Valsa mali
Verticillium dahliae
Ustilaginoidea virens
Colletotrichum fructicola
Colletotrichum fructicola
Botrytis cinerea
Sclerotinia sclerotiorum
Verticillium dahliae
Valsa mali
Pyricularia oryzae
Valsa mali
Colletotrichum fructicola
Botrytis cinerea
Sclerotinia sclerotiorum
Sclerotinia sclerotiorum
Botrytis cinerea
Botrytis cinerea

C

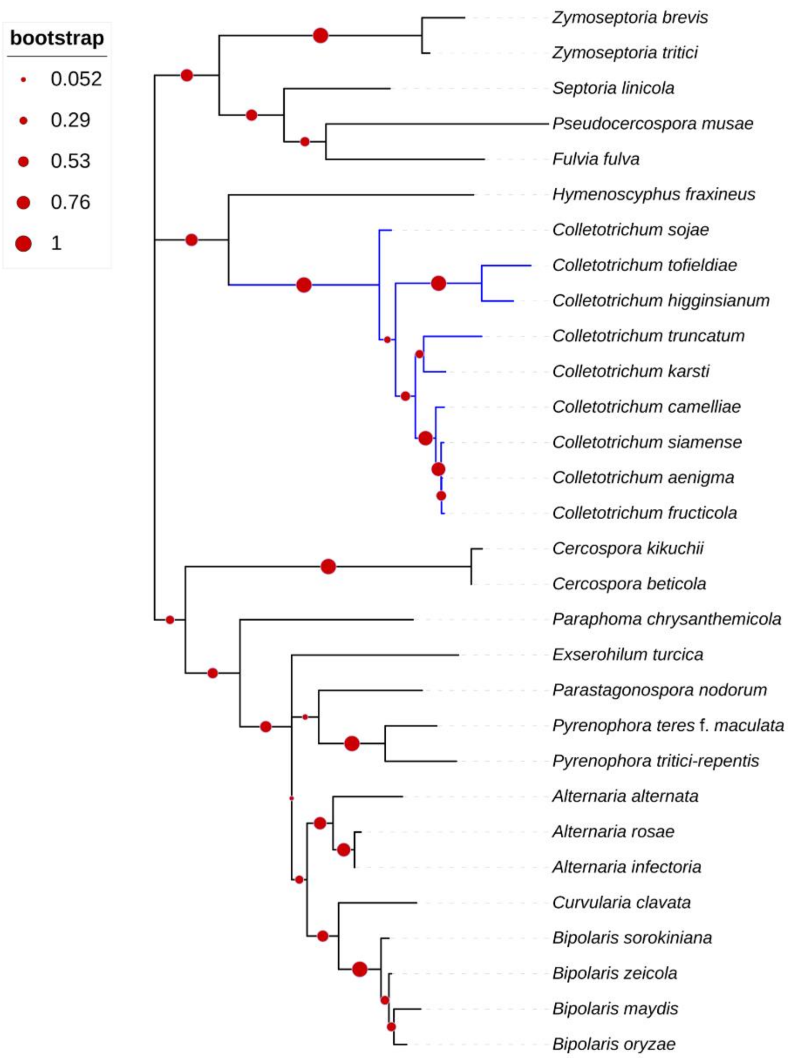
bootstrap
0.052
0.29
0.53
0.76
1
*Zymoseptoria brevis*
*Zymoseptoria tritici*
*Septoria linicola*
*Pseudocercospora musae*
*Fulvia fulva*
*Hymenoscyphus fraxineus*
*Colletotrichum sojae*
*Colletotrichum tofieldiae*
*Colletotrichum higginsianum*
*Colletotrichum truncatum*
*Colletotrichum karsti*
*Colletotrichum camelliae*
*Colletotrichum siamense*
*Colletotrichum aenigma*
*Colletotrichum fructicola*
*Cercospora kikuchii*
*Cercospora beticola*
*Paraphoma chrysanthemicola*
*Exserohilum turcica*
*Parastagonospora nodorum*
*Pyrenophora teres* f. *maculata*
*Pyrenophora tritici-repentis*
*Alternaria alternata*
*Alternaria rosae*
*Alternaria infectoria*
*Curvularia clavata*
*Bipolaris sorokiniana*
*Bipolaris zeicola*
*Bipolaris maydis*
*Bipolaris oryzae*

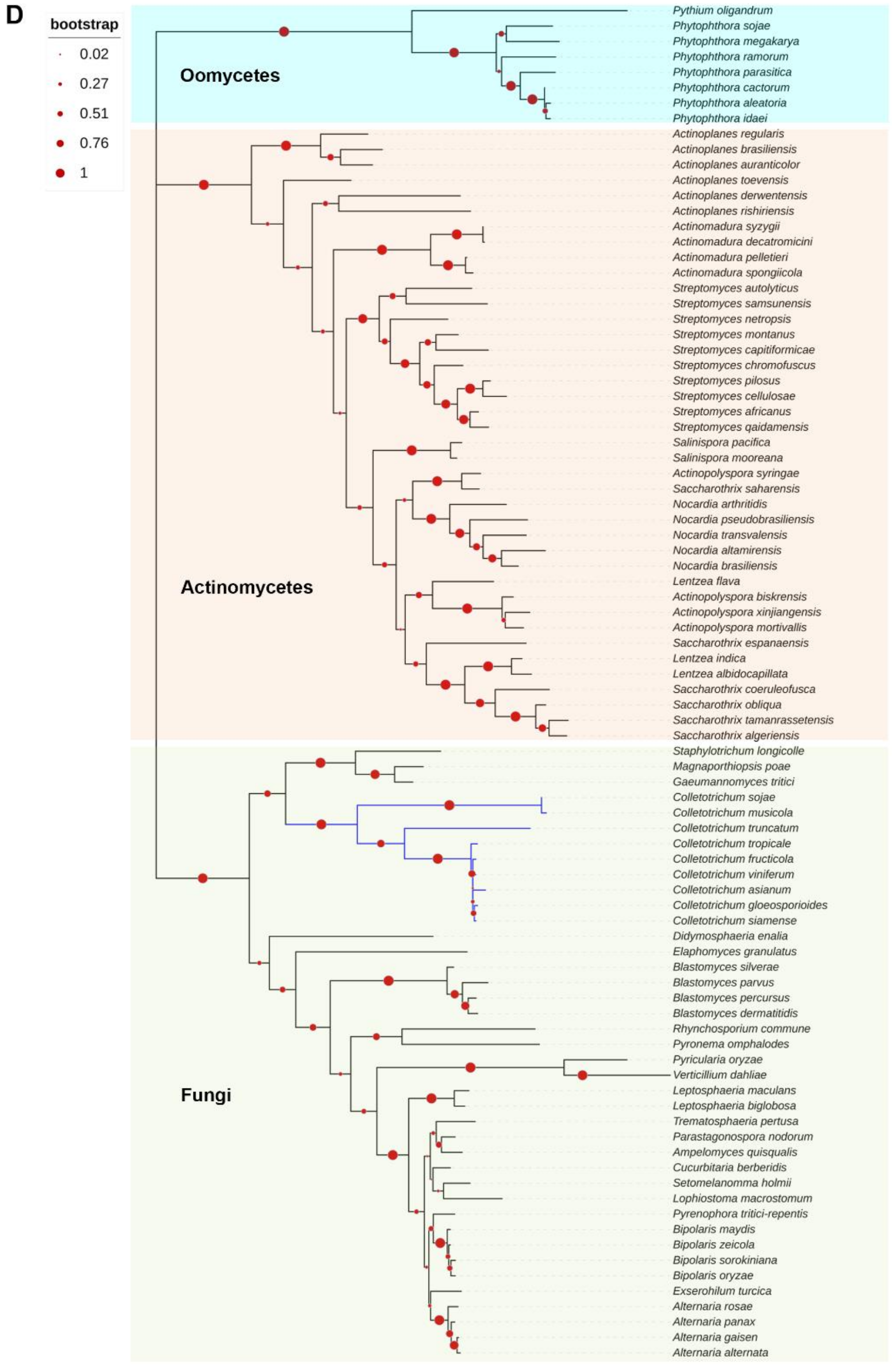


**Figure S4.** The phylogeny of the four core effectors. The trees for CfCE4 (**A**), CfCE25 (**B**), CfCE61 (**C**) and CfCE66 (**D**) were constructed with the maximum-likelihood method. Bootstrap percentage support for each branch is indicated.

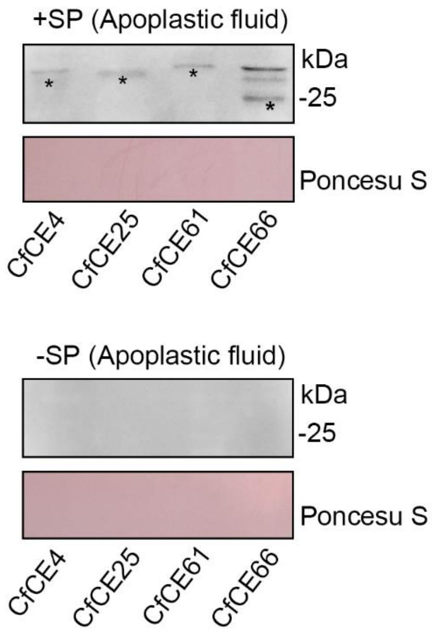


**Figure S4.** The core effectors are accumulated in apoplastic fluid of *N. benthamiana*. The full-length CfCE4, CfCE25, CfCE61 and CfCE66 (+SP), and their SP-deleted versions (-SP) were transiently in *N. benthamiana* via agroinfiltration. The apoplastic fluid in the leaves was extracted and subjected to western blotting with anti-Flag antibody. Black asterisks indicate the bans with expected size. Total proteins were stained with Ponceau S to check the quality of apoplastic fluid.
